# Ruptured Aneurysm in Complete Duplication of the Posterior Cerebral Artery: Successful Treatment With Endovascular Coiling

**DOI:** 10.7759/cureus.99964

**Published:** 2025-12-23

**Authors:** I-Chen Wu, Ching-Chung Ko, Te-Chang Wu, Yu-Kun Tsui

**Affiliations:** 1 Department of Radiology, Chi Mei Medical Center, Tainan, TWN

**Keywords:** complete duplication of the posterior cerebral artery, endovascular coiling, fetal-type posterior cerebral artery, posterior communicating artery aneurysm, ruptured intracerebral aneurysm

## Abstract

The fetal posterior cerebral artery (FPCA) variant is rare, and among these, complete duplication of the posterior cerebral artery (PCA) represents the rarest form. In this case report, we describe a 39-year-old woman with an aneurysm arising from complete duplication of the PCA. The aneurysm was successfully occluded while preserving FPCA patency using endovascular coiling with the double-microcatheter technique, and no recurrence of the aneurysm was observed on brain magnetic resonance angiography (MRA) at six-month follow-up. To the best of our knowledge, this is the first reported case of an aneurysm arising from complete duplication of the PCA that was successfully treated by endovascular coiling. We highlight the anatomical characteristics of complete duplication of the PCA, the utility of multimodal imaging in identifying FPCA variant, and the need to preserve the FPCA during aneurysm treatment to prevent PCA territory infarction.

## Introduction

The fetal posterior cerebral artery (FPCA) is an embryological remnant identified in approximately 11%-46% of adults and encompasses a group of developmental variants of the posterior cerebral artery (PCA) [1]. An absent or hypoplastic P1 segment with a prominent posterior communicating artery (PCoA) is relatively common in clinical practice, whereas other variants, such as a true FPCA and complete duplication of the PCA, are rare [2-4]. Among these variants, complete duplication of the PCA is the rarest form. Both true FPCA and complete duplication of the PCA involve two independent posterior cerebral arteries, albeit with subtle differences in their anatomical configuration. True FPCA is characterized by persistence of a large primitive anterior choroidal artery (AChA) arising from the internal carotid artery (ICA), coexisting with the classical posterior cerebral artery originating from the basilar trunk. In contrast, complete duplication of the PCA features a normal AChA, a fetal-type PCoA arising from the ICA, and the classical PCA originating from the basilar trunk. In individuals with the FPCA variant, the PCA territory is primarily supplied by the anterior circulation rather than the posterior circulation [2]. Consequently, thromboembolic events originating from the ICA, or inadvertent compromise of the FPCA during cerebrovascular interventions, may result in infarction within the PCA territory [5,6]. When treating a PCoA aneurysm, patients without the FPCA variant may tolerate sacrifice of the PCoA without complications. However, in cases with an FPCA variant, the FPCA must be fully preserved to prevent infarction in the PCA territory. In this case report, we describe a ruptured aneurysm associated with complete duplication of the PCA that was successfully treated with endovascular coiling while preserving the FPCA, a scenario not previously reported. We highlight the exceptionally rare complete duplication of the PCA, the utility of multimodal imaging in identifying FPCA variants, and the need to preserve the FPCA during aneurysm treatment to prevent PCA territory infarction.

## Case presentation

**Figure 1:**
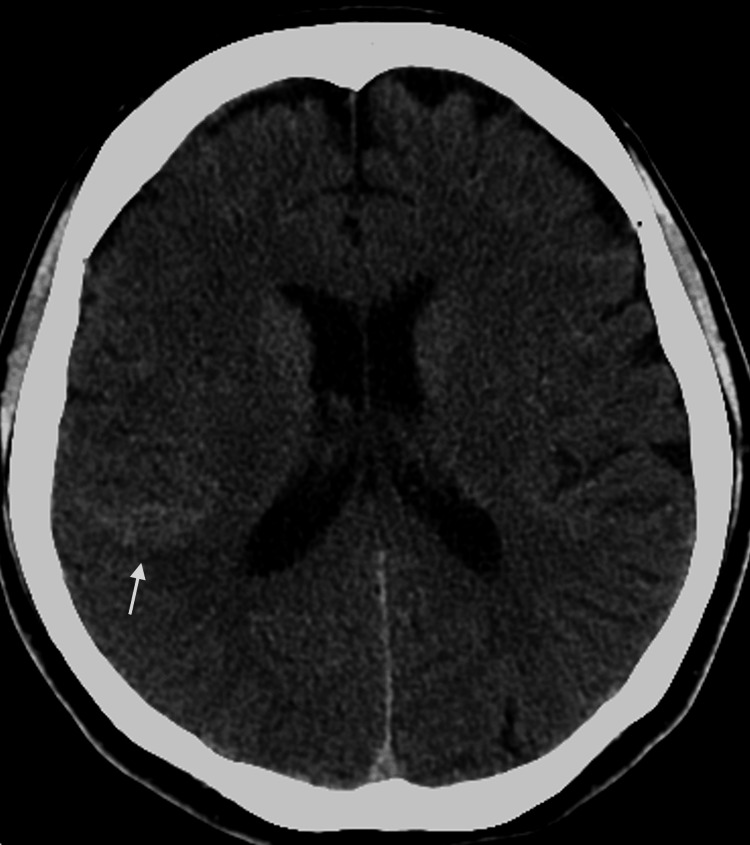
Non-contrast brain CT Non-contrast brain CT demonstrates subarachnoid hemorrhage predominantly located in the right frontotemporal sulci (arrow). CT: Computed tomography

**Figure 2:**
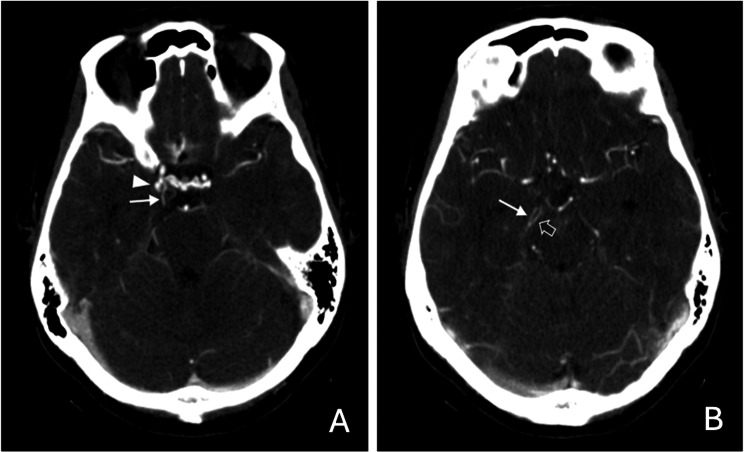
Consecutive axial CTA sections of the brain (A,B) The images show an aneurysm (arrowhead in A) at the junction of the right ICA and PCoA (arrows in A and B). The right PCoA extends posteriorly as a prominent vessel running parallel to the classical right PCA arising from the basilar trunk (open arrow in B) toward the occipital lobe. CTA: Computed tomography angiography, ICA: Internal carotid artery, PCoA: Posterior communicating artery, PCA: Posterior cerebral artery

A 39-year-old woman with no significant medical or family history, who was a non-smoker and did not consume alcohol, presented with a sudden, severe thunderclap headache unlike any she had experienced before. Non-contrast brain computed tomography (CT) revealed subarachnoid hemorrhage (SAH) (Figure 1). On neurological examination, she had a Glasgow Coma Scale score of E4V5M6, with no neurological deficits, corresponding to a Hunt and Hess grade II and a modified Rankin Scale (mRS) score of 2. Computed tomographic angiography (CTA) demonstrated an aneurysm at the junction of the right ICA and the PCoA. The right PCoA extended posteriorly as a prominent vessel running parallel to the classical right PCA arising from the basilar trunk (Figure 2).

**Figure 3:**
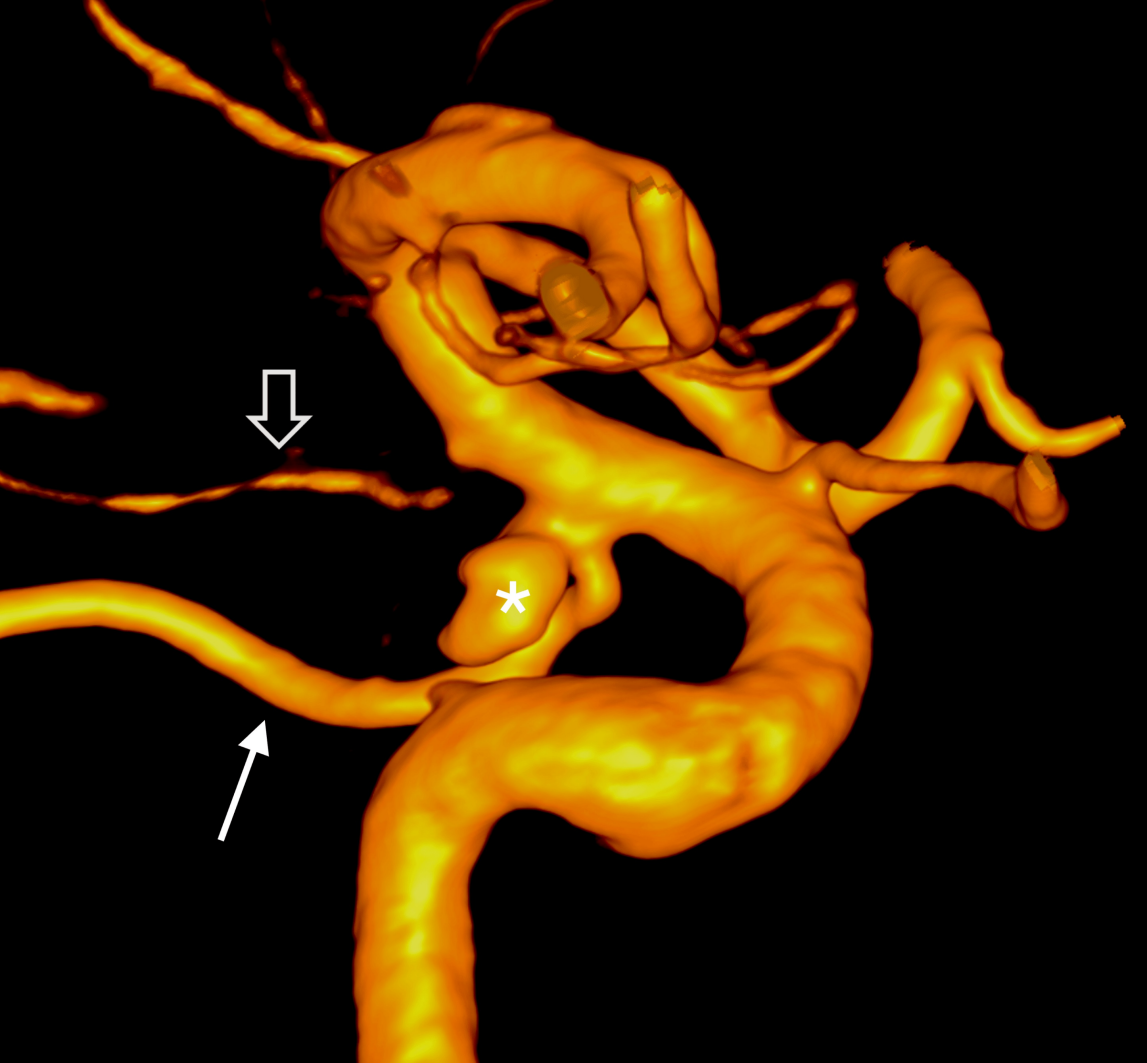
Three-dimensional reconstruction of rotational angiography demonstrating the right ICA and PCoA A wide-neck aneurysm (asterisk) is located at the orifice of the PCoA (arrow); a normal AChA (open arrow) is noted. ICA: Internal carotid artery, PCoA: Posterior communicating artery, AChA: Anterior choroidal artery

This configuration differs from a typical hyperplastic PCoA. Subsequent digital subtraction angiography (DSA) confirmed a wide-neck aneurysm, and the normal anterior choroidal artery (AChA) was clearly delineated on three-dimensional rotational angiography (Figure 3).

**Figure 4:**
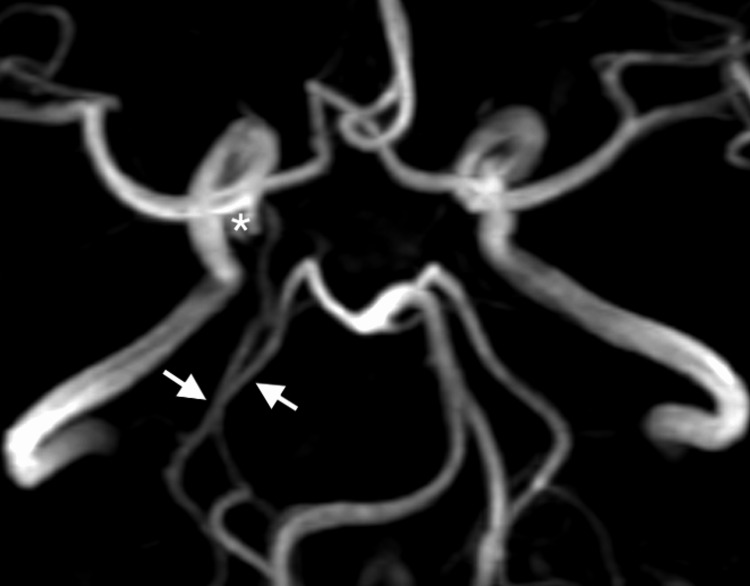
3T brain MRA The image shows right complete duplication of the PCA (arrows) and an aneurysm (asterisk) located at the base of FPCA arising from the ICA. MRA: Magnetic resonance angiography, PCA: Posterior cerebral artery, FPCA: Fetal posterior cerebral artery, ICA: Internal carotid artery

During DSA of the posterior circulation, a perfusion defect was observed in the right PCA territory. Both the CTA and DSA findings raised suspicion for the presence of an FPCA variant. As further treatment was being considered, magnetic resonance angiography (MRA) was performed to better delineate the vascular anatomy, revealing an uncommon complete duplication of the right PCA. One PCA originated from the basilar trunk and supplied the parieto-occipital region, while the other arose from the ICA as an FPCA and supplied the temporal region of the PCA territory. The aneurysm was located at the base of the FPCA, arising from the ICA (Figure 4). 

**Figure 5:**
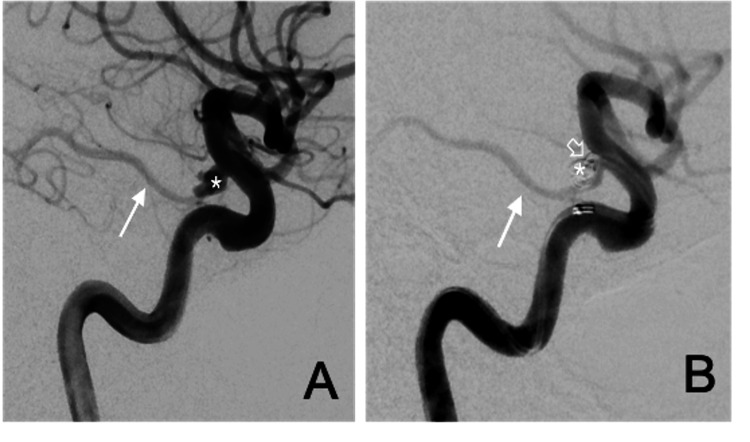
Lateral projection right internal carotid arteriograms obtained at baseline (A) and after endovascular coiling (B) (A) The aneurysm (asterisk) is located at the base of the FPCA (arrow) arising from the ICA. (B) Near-total obliteration of the aneurysm (asterisk) with a shallow triangular residual neck (open arrow) was observed after the procedure. The FPCA (arrow) remains well preserved. FPCA: Fetal posterior cerebral artery, ICA: Internal carotid artery

**Figure 6:**
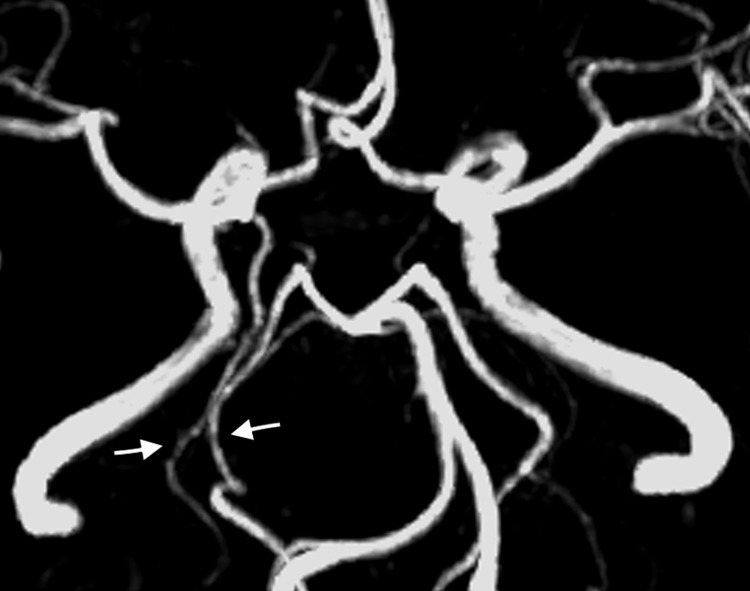
Six-month follow-up brain MRA MRA demonstrates preserved right complete duplication of the PCA (arrows) and no recurrence of the aneurysm. MRA: Magnetic resonance angiography, PCA: Posterior cerebral artery

The wide-neck aneurysm, measuring approximately 4.6 mm in dome height and 3.5 mm at the neck with a dome-to-neck ratio of less than 2, was treated by endovascular coiling. Under general anesthesia, a 6-French guiding catheter was advanced into the right ICA. Using the double microcatheter technique, two microcatheters (Excelsior SL-10 and Headway-17) were navigated into the aneurysmal sac. A framing coil (target helical 4 mm × 6 cm) was first deployed without compromising adjacent normal vessels, followed by sequential packing with smaller coils until obliteration of the aneurysmal cavity was achieved. The post-procedure right internal carotid angiogram showed near-total obliteration of the aneurysm with a 1 mm shallow triangular residual neck. The FPCA was well preserved (Figure 5). As the patient exhibited no hydrocephalus, neither an external ventricular drain (EVD) nor intracranial pressure (ICP) monitoring was required. She received nimodipine and valproate sodium as part of post-SAH medical management. No antiplatelet therapy was administered, as no stent placement was performed. The patient was discharged 14 days after treatment with a modified Rankin Scale (mRS) score of 1, indicating only mild residual headache without neurological deficits. Brain MRA follow-up was performed every six months after discharge, and the corresponding follow-up MRA showed no recurrence of the aneurysm (Figure 6). 

## Discussion

The FPCA variant is an embryological remnant that develops around the choroidal stage, approximately 5 weeks into gestation, and has been classified in numerous studies [1]. In one study, the FPCA was classified into four distinct variants: full FPCA (absent or non-visualized P1), partial FPCA (P1 smaller than the PCoA), intermediate FPCA (P1 and PCoA of similar caliber), and true FPCA, also called duplication of the PCA, in which two independent PCAs arise, with one dominant PCA originating from a persistent large primitive AChA, and the other developing normally from the basilar trunk. In the true FPCA, the persistent primitive AChA supplies not only the AChA territory but also part of the PCA territory [3,15,16]. Another study described a similar variant, called complete duplication of the PCA, characterized by two PCAs arising separately from the ICA and the basilar trunk, accompanied by a normal AChA. It is the rarest FPCA variant, with an estimated incidence of approximately 0.04% among all FPCA variants [4,17]. 

In individuals with a normally configured circle of Willis, sacrifice of the PCoA during endovascular treatment of a PCoA aneurysm is generally considered safe [18]. However, in patients with FPCA variant, such an approach may result in ischemic complications during endovascular treatment [6,18,19]. The treatment of PCoA aneurysms associated with FPCA is technically challenging because the FPCA must be completely preserved. Several studies have suggested that selecting appropriate adjunctive techniques according to the aneurysm’s morphology and location may help preserve the FPCA while minimizing the risk of ischemic complications, including the double microcatheter technique, balloon-remodeling technique, stent-assisted coiling, Y-stenting technique, and flow-diverting stent deployment [5,9]. We reviewed previous studies on the management of FPCA aneurysms, focusing on strategies aimed at preserving the FPCA (Table 1). In the reviewed studies, most reported FPCA variants involved aplasia or hypoplasia of the P1 segment. One study described a duplicated PCA without a normal AChA, while two studies did not specify the variant type. No study has reported complete duplication of the PCA. Regardless of the FPCA variant, preservation of the FPCA is generally prioritized during treatment, whether by surgical clipping or endovascular coiling.

**Table 1:** Literature review of FPCA aneurysms treated with preservation of FPCA patency CTA: Computed tomography angiography, DSA: Digital subtraction angiography, MRI: Magnetic resonance imaging, FPCA: Fetal posterior cerebral artery, ICA: Internal carotid artery, PCoA: Posterior communicating artery, AChA: Anterior choroidal artery

Author	Year	Number of cases (N)	Imaging modality	FPCA variant	Location of the aneurysm	Treatment	Patency of FPCA after treatment
Nomura et al. [7]	2025	1	CTA, DSA	P1 segment aplasia	PCoA	Endovascular coiling with stent assistance	Preserved
Ahmed et al. [8]	2024	1	CTA, DSA	Duplicated FPCA without normal AChA	PCoA	Endovascular coiling	Preserved
Fuga et al. [9]	2023	19	DSA	P1 segment aplasia	PCoA	Endovascular coiling with stent assistance (n = 8) or without stent assistance (n = 11)	Preserved
Cavasin et al. [10]	2022	1	CTA, DSA	P1 segment hypoplasia	PCoA	Endovascular coiling	Preserved
Nariai et al. [11]	2022	6	DSA	-	PCoA	Endovascular coiling with balloon assistance	Preserved
Tanabe et al. [12]	2021	8	DSA	P1 segment hypoplasia	PCoA	Endovascular coiling with stent assistance	Preserved
Raper et al. [13]	2020	1	MRI, DSA	-	PCoA	Endovascular coiling with stent assistance	Preserved
Chen et al. [5]	2015	9	CTA ,MRI, DSA	P1 segment aplasia	PCoA	Endovascular coiling with balloon, stent, or double microcatheter assistance	Preserved
Zadaet al. [14]	2008	30	DSA	P1 segment aplasia or hypoplasia	PCoA	Surgical clipping (n = 24) and endovascular coiling (n = 6)	Preserved (endovascular n = 6; surgical n = 21), occluded (surgical n = 1), not assessed (n = 2)

To the best of our knowledge, this is the first reported case of endovascular coiling for an aneurysm arising from a rare complete duplication of the PCA. Our case demonstrated a normal AChA and two distinct PCAs originating separately from the ICA and the basilar trunk, representing a complete duplication of the PCA. The aneurysm was located at the base of the FPCA, arising from the ICA, and was successfully occluded while preserving FPCA patency using endovascular coiling with the double-microcatheter technique. This technique was selected because it is recommended for wide-neck ruptured aneurysms, and the operator was experienced with this approach [6]. In this case, when CTA and DSA were insufficient to definitively characterize the FPCA variant, MRA provided valuable, noninvasive visualization of the variant and its anatomical relationship to the aneurysm. Accordingly, we suggest employing multimodal imaging prior to treatment of a PCoA aneurysm to ensure precise pre-procedural identification of an FPCA variant and to guide the selection of the most appropriate treatment strategy [8,20].

## Conclusions

This case describes a ruptured aneurysm arising from an extremely rare complete duplication of the PCA that was successfully treated with endovascular coiling while preserving the FPCA. Follow-up imaging demonstrated no aneurysm recurrence, and the patient achieved an excellent clinical outcome. Preservation of the FPCA during the treatment of a PCoA aneurysm is essential, as inadvertent occlusion may result in infarction. Therefore, accurate identification of an FPCA variant before treatment is critical. The use of multimodal imaging, including CTA, DSA, and especially MRA, facilitates a comprehensive pre-procedural vascular assessment. However, as this report describes a single case, the conclusions should be considered illustrative rather than broadly generalizable. Further studies are needed to confirm these observations in larger patient cohorts.
